# Integrating the lactulose-mannitol test for intestinal permeability with untargeted metabolomics for drug monitoring through dual liquid chromatography-mass spectrometry

**DOI:** 10.1007/s00216-025-05790-7

**Published:** 2025-02-27

**Authors:** Felina Hildebrand, Cemre Cukaci, Harald Schoeny, Christoph Baumgartinger, Bruno Stelzer, Matteo Spedicato, Tobias Frey, Martina Catani, Klaus Schmetterer, Richard Frey, Gunda Koellensperger

**Affiliations:** 1 Department of Analytical Chemistry, University of Vienna, Waehringer Str. 38, 1090 Vienna, Austria; 2 Vienna Doctoral School in Chemistry (DoSChem), University of Vienna, Waehringer Str. 42, 1090 Vienna, Austria; 3 Division of General Psychiatry, Department of Psychiatry and Psychotherapy, Medizinische Universität Wien/Medical University of Vienna, Vienna, Österreich; 4 Comprehensive Center for Clinical Neurosciences and Mental Health, Medizinische Universität Wien/Medical University of Vienna, Vienna, Österreich; 5 Department of Chemical, Pharmaceutical and Agricultural Sciences – DOCPAS, University of Ferrara, Via L.Borsari 46, 44121 Ferrara, Italy; 6 Department of Laboratory Medicine, Medical University of Vienna, Waehringer Guertel 18-20, 1090 Vienna, Austria; 7 Vienna Metabolomics Center (VIME), University of Vienna, Althanstr. 14, 1090 Vienna, Austria

**Keywords:** Intestinal permeability testing, Dual sugar test, Drug monitoring, Dual LC–MS, Clinical/biomedical analysis

## Abstract

In this study, we developed a customized high-resolution mass spectrometry metabolomics workflow integrating the dual sugar test employing lactulose and mannitol as test probes for intestinal permeability assessment with untargeted screening of small molecules. Urine samples were collected from patients with major depression and healthy controls as part of a clinical study at the psychiatric department. Using a dual injection/dual chromatography setup, the test probes were quantified by hydrophilic interaction liquid chromatography (HILIC) in a targeted assay, while drugs and their metabolites were profiled in an untargeted manner by reversed-phase separation. Rigorous method development and validation allowed for selective separation of sugar isomers and consequently accurate quantification of lactulose and mannitol in urine. Internal standardization with compound specific stable isotope-labeled standards enabled excellent analytical figures of merit such as high recoveries, precision (< 5%), and working range (5 orders of magnitude). Within one analytical run, intestinal permeability was assessed together with drugs and their metabolites, allowing to screen for confounding drugs and patient compliance to the therapeutic scheme.

**Graphical Abstract:**

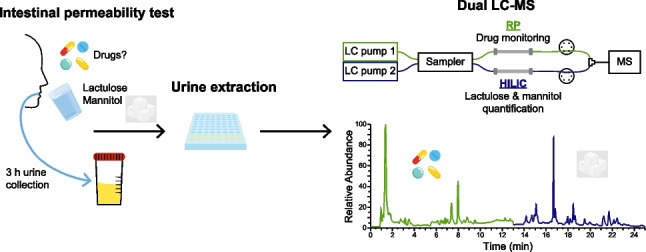

**Supplementary Information:**

The online version contains supplementary material available at 10.1007/s00216-025-05790-7.

## Introduction

Liquid chromatography (LC) coupled to mass spectrometry (MS) is an essential platform in clinical analysis [1]. The last decades have seen a resurgence of research on metabolism. Metabolomics empowers to assess the metabolic phenotype of a biological specimen, directly linked to disease phenotypes [2]. This direct link supports biomarker research, diagnostics and research on disease etiology and mechanisms [1, 3]. Especially, untargeted omics type of analysis offers indispensable opportunities for clinical research nowadays. Several targeted metabolite assays are part of clinical diagnostic routines (e.g., newborn screening, metabolic disorders) [4, 5]. Stringent guidelines such as in vitro diagnostic guidelines provide the regulatory frame for their routine application [6, 7]. There are many reasons to combine both targeted and untargeted analysis strategies into one clinical study. For example, in biomarker research, the study design includes an analytical validation of biomarkers, discovered by untargeted metabolomics and confirmed by targeted analysis [3]. High-resolution mass spectrometry supports both quantification and identification by accurate mass and MS2 experiments [8]. As a consequence, merged workflows were successfully proposed, covering relative quantification and metabolite identification together with the absolute quantification of a subset of metabolites. This way, customized approaches with tailored selectivity and accuracy can be designed to increase analytical throughput [9–11].

In this work, another prime example emphasizing the value of merged strategies will be elaborated, i.e., controlling and discovering confounding factors through untargeted analysis integrated with a targeted assay in clinical research. The importance of considering confounding factors that could otherwise lead to misinterpretation has been extensively discussed [12–16]. This study addresses an intervention-based test for intestinal permeability, which describes the capability of small molecules to pass through the gastrointestinal mucosa by unmediated diffusion. It is a highly dynamic process that can be influenced by many factors [17]. Therefore, the assessment of confounding variables affecting the association between intervention and outcome is of utmost importance. The capability of drugs to increase intestinal permeability, particularly non-steroidal anti-inflammatory drugs (NSAIDs), has been well studied, highlighting the importance of considering drug effects in such tests [18, 19].

Above all, despite the long-established practice of testing for intestinal permeability, the procedure is not standardized [20, 21]. A variety of test probes are administered orally in different quantities, and urine is collected over varying time periods [17, 22]. One common test approach is the dual sugar test utilizing the different absorption of a disaccharide and a monosaccharide by the intestinal barrier. Two commonly employed sugars in the test are lactulose, a synthetic disaccharide that is not hydrolyzed in the small intestine and thus undergoes minimal absorption, and the sugar alcohol mannitol as monosaccharide. However, the test can also be conducted using different sugar combinations like lactulose together with rhamnose. It is essential that the absorbed amount after penetration of the mucosa for both sugars is directly reflected in the urine, which is the biofluid analyzed for intestinal permeability testing [17, 22]. The sugars are absorbed along the crypt-villus axis of the small intestine. The smaller sugar probe, mannitol, is capable to pass through the smaller, accessible, and numerous tight junctions of the villus tip. The larger probe, lactulose, is only able to pass through the larger, less accessible, and less numerous pores at the crypt base, resulting in limited penetration of the mucosa under normal conditions [22]. The urinary mannitol excretion is an indicator of the absorptive surface area, while the urinary lactulose excretion directly reflects intestinal permeability [23]. Eventually, the lactulose:mannitol ratio (LMR) is considered as the measure of the intestinal permeability as it, compared to lactulose excretion alone, and also accounts for non-mucosal factors (e.g., gastric emptying, intestinal transit time, renal clearance, and incomplete urine recovery), which can influence the amount of sugar excreted into urine [22–25]. Intestinal permeability has been described for various conditions such as inflammatory bowel disease [26], Crohn’s disease [27], and irritable bowel syndrome [28]. A dysfunction of the barrier between the gastrointestinal tract and the bloodstream can result in exposure to potentially pro-inflammatory molecules, thereby accelerating disease progression [17].

This study evaluates several chromatographic separation conditions for sugar and sugar alcohol isomer separation and presents a fit-for-purpose merged method that integrates intestinal permeability testing with drug monitoring in a single LC–MS run using a dual LC setup. The sugars utilized for intestinal permeability testing, i.e., lactulose and mannitol, are quantified with stable isotope-labeled (SIL) internal standards by hydrophilic interaction liquid chromatography (HILIC). Potential confounding drugs, such as NSAIDs, which can influence the intestinal permeability test results [18, 19], were addressed in parallel using a dual chromatography approach. Thus, while absolute quantification of the sugars was performed by HILIC-MS, the drug screening was optimized on parallel reversed-phase (RP) chromatography, performed within one analytical run.

This integrated methodology was showcased in a small cohort of patients with major depressive disorder undergoing intestinal permeability testing. The strategy successfully supported the analysis of intestinal permeability, screening for confounding drugs, and the assessment of participants’ drug compliance. This approach can pave the way for linking intestinal permeability testing with metabolic activity and drug screening.

## Material and methods

### Study cohort

The study cohort consisted of patients diagnosed with major depressive disorder, with a Montgomery–Åsberg Depression Rating Scale (MADRS) score of 20 or higher, corresponding to a moderate (scores 20–34) or severe (scores 35–60) treatment-resistant depressive episode under psychotropic medication [29, 30]. Patients were admitted to the Vienna General Hospital, Medical University Vienna, Department of Psychiatry and Psychotherapy, for inpatient treatment. Ethical approval was granted by the ethics committee of the Medical University of Vienna (EK-Nr: 1882/2020). In this work, five patient samples and five control samples were analyzed as a proof of principle. Table S1 provides information on sex and age. Each group comprised two females and three males with one person < 23 years and one person > 60 years.

### Intestinal permeability test and urine sample collection

For the assessment of intestinal permeability, patients were orally administered 200 mL of a water solution containing 10 g of lactulose and 5 g of mannitol and an additional 400 mL of water on an empty stomach in the morning after an overnight fast. Urine was collected over a 3-h period, during which patients were not allowed to eat or drink. After that, the total urine volume was measured, and 0.5 mL aliquots were stored at − 80 °C.

### Creatinine quantification in urine

Creatinine levels in urine were determined using the standard routine diagnostic test Creatinine Jaffe Gen.2 on a cobas e801 analyzer (both Roche) at the diagnostic laboratory at the Department of Laboratory Medicine, Medical University of Vienna.

### LC method development for targeted measurement of lactulose and mannitol

LC method development for the targeted measurement of lactulose and mannitol was focused on the separation of both targeted compounds from their isomers, which can be present in urine. An equimolar sugar multi-component standard mix containing mannitol, lactulose, galactitol, sorbitol, melibiose, cellobiose, maltose, lactose, sucrose, and isomaltose, as well as two-compound mixes of mannitol and lactulose, galactitol and cellobiose, and sorbitol and sucrose were prepared. All other compounds were additionally prepared as single standards.

To achieve the best possible separation, different stationary and mobile phases were tested. All stationary phases were tested using different LC conditions (column temperature, flow, and gradient). In Table 1, the stationary phases are listed together with the best performing mobile phases and LC conditions. The sugar multi-component standard mix as well as the two-compound mixes and single standards were injected at concentrations ranging between of 0.5 and 5 µM, and the injection volume was 5 µL. For the HILIC separations, the mixes were dissolved in 80:20 ACN/H_2_O (v/v), and for the mixed mode and porous graphitic carbon (PGC) column in 100% H_2_O.

**Table 1:** LC columns with corresponding mobile phases, LC conditions and gradients tested for sugar isomer separation, and targeted measurement of lactulose and mannitol

Method number	Stationary phase	LC column	Mobile phases	LC conditions	Gradient
1	HILIC (sulfobetaine)	Atlantis PREMIER BEH Z-HILIC (2.1 × 150 mm, 1.7 μm, Waters)	Eluent A: 100% H_2_O; Eluent B: 90:10 ACN/H_2_O; Both with 15 mM ammonium bicarbonate at pH 9.0	Column temperature: 40 °C Flow: 0.3 mL min^−1^	0–2 min decrease to 85% B, 2–7 min 85% B, 7–10 min decrease to 40% B, 10–11 min 40% B, 11–15 min re-equilibration at 100% B
2	HILIC (amide)	ACQUITY UPLC BEH Amide (2.1 × 150 mm, 1.7 μm, Waters)	Eluent A: 100% H_2_O; Eluent B: 90:10 ACN/H_2_O; Both with 10 mM ammonium bicarbonate at pH 9.0	Column temperature: 40 °C Flow: 0.3 mL min^−1^	0–3 min decrease to 85% B, 3–5 min 85% B, 5–10 min decrease to 50% B, 10–12 min 50% B, 12–15 min re-equilibration at 100% B
3	Mixed mode (reversed-phase and anion exchange)	Atlantis PREMIER BEH C18 AX (2.1 × 150 mm, 1.7 μm, Waters)	Eluent A: 100% H_2_O; Eluent B: 90:10 ACN/H_2_O; 15 mM ammonium bicarbonate at pH 9.0	Column temperature: 40 °C Flow: 0.25 mL min^−1^	0–1 min 0% B, 1–15 min increase to 100% B, 15–16 min 100% B, 16–21 min re-equilibration at 0% B
4	Porous Graphitic Carbon	Hypercarb™ Porous Graphitic Carbon (2.1 × 150 mm, 3 μm, Thermo Fisher)	Eluent A: 100% H_2_O; Eluent B: 90:10 ACN/H_2_O; Both with 0.1% NH_4_OH	Column temperature: 40 °C Flow: 0.5 mL min^−1^	0–3 min 0% B, 3–9 min increase to 75% B, 9–10 min 75% B, 10–15 min re-equilibration at 0% B

The instrumental platforms used for LC method development were either a Vanquish Duo UHPLC system or Vanquish Horizon UPLC with MS detection by Q Exactive HF Orbitrap or Orbitrap ID-X Tribrid Mass Spectrometer (all Thermo Fisher Scientific).

Chromatographic resolution (Eq. 1: LC peak resolution, where *R*_*S*_ is the peak resolution, *t*_*R*_ is the retention time, and *w*_*b*_ is the peak baseline width) was calculated after manual peak detection within FreeStyle (version 1.8 SP2 QF1, Thermo Fisher Scientific).

1 $${R}_{S}=2\frac{{t}_{R2}-{t}_{R1}}{{w}_{b1}+{w}_{b2}}$$

### Standard and sample preparation

To evaluate, which concentrations of sugar isomers interfere with lactulose and mannitol and hamper accurate quantification, lactulose and mannitol standard mixes at different concentrations (0.075 µM, 0.15 µM, 1.5 µM, and 20 µM) were spiked with sugar their isomers (sorbitol isomeric to mannitol and sucrose, lactose, and maltose isomeric to lactulose) also at different concentrations (0.05 µM, 0.5 µM, 2.5 µM, and 5 µM). The 16 mixes were spiked with 1 µM of lactulose-^13^C12 and 0.5 µM of D-mannitol-^13^C6. The final solvent composition for LC–MS measurement was 80:20 ACN/water (v/v). Further information on the methodical procedure for the evaluation of isomer interferences can be found in the Electronic Supplementary Material.

Sample preparation for the quantification of lactulose and mannitol was either done in 2-mL microcentrifugation tubes or in a down-scaled manner in 96-well plates. Extraction was done on ice with 80% methanol and spiking of stable isotope labeled (SIL) internal standards (lactulose-^13^C12 and D-mannitol-^13^C6). Samples were diluted in 80:20 ACN/water (v/v) for HILIC measurements. For untargeted measurements, the down-scaled extraction procedure was followed without spiking of SIL internal standards and dilution of samples in water for injection on RP. Detailed extraction procedures are described in the Electronic Supplementary Material.

For quantification of lactulose and mannitol calibration curves at different standard concentrations spiked with SIL internal standards were prepared in 80:20 ACN/water (v/v) (Electronic Supplementary Material).

### LC–MS methods for method validation and urine measurement

Two different LC–MS measurements were performed using a Vanquish Duo UHPLC (Thermo Fisher Scientific) coupled to different MS instruments: (1) stand-alone HILIC coupled to Orbitrap ID-X Tribrid mass spectrometer (Thermo Fisher Scientific) and (2) dual LC coupled to a Q Exactive HF™ quadrupole-Orbitrap mass spectrometer (Thermo Fisher Scientific).

For the first setup, HILIC separation was achieved as described previously (Table 1, method 1) with a prolonged re-equilibration time of 5 min. The injection volume was 5 µL of samples dissolved in 80% ACN. The autosampler and column temperature were constantly maintained at 6 °C and 40 °C, respectively. Ionization after LC separation was achieved using heated electrospray ionization (HESI) with the following parameters: spray voltage of 3.5 kV (positive mode) and 2.3 kV (negative mode), sheath gas 40, auxiliary gas 3, capillary temperature of 300 °C, vaporizer temperature of 200 °C, and a RF lens value of 40%. Data acquisition was done by polarity switching in MS1 mode with a resolution of 60,000, a scan range of 60–900 m*/z*, a maximum injection time of 100 ms, and a normalized AGC target of 33%.

For the second setup, a dual LC setup was used to merge targeted measurements using HILIC and untargeted measurements using RP in parallel [31]. RP separation was achieved using an ACQUITY UPLC HSS T3 C18 column (2.1 × 150 mm, 1.8 μm, Waters) equipped with an ACQUITY UPLC HSS T3 VanGuard Pre-column (2.1 × 5 mm, 1.8 μm, Waters). Mobile phases were water with 0.1% formic acid as eluent A and MeOH with 0.1% formic acid as eluent B. The flow rate was 0.3 mL min^−1^ during gradient elution and the following gradient was run: 0–3 min 0% B, 3–10 min ramp to 100% B, 10–15 min 100% B, and 15–25 min re-equilibration at 0% B. During re-equilibration, the flow was reduced to 0.05 mL min^−1^ from 18 to 22 min and then increased again to 0.3 mL min^−1^ from minute 22 to 24. The injection volume was 5 µL of 100% aqueous samples. HILIC separation was achieved as described previously (Table 1, method 1). However, re-equilibration was from 0 to 13 min during the measurement of RP. The HILIC gradient started after 13 min. After the 11-min-long gradient, at minute 24, the column re-equilibration was started. During re-equilibration, the flow was reduced to 0.1 mL min^−1^ from 2 to 8 min and then increased again to 0.3 mL min^−1^ from minute 8 to 10. The injection volume was 5 µL of samples dissolved in 80% ACN. The autosampler and column temperature were constantly maintained at 6 °C and 40 °C, respectively. For the dual LC measurement, the RP was coupled to MS for the first 13 min. Afterwards, both valves were switched and the HILIC was coupled to MS. After 24.9 min, the valves were switched back to the initial position. The duration of a dual LC run was in total 25 min. MS detection was done using full MS acquisition combined with data-dependent acquisition (DDA). Ion source settings for HESI were the following: spray voltage of 3.1 kV, capillary temperature of 300 °C, sheath gas flow rate of 60, aux gas flow rate of 25, sweep gas flow rate of 2, heater temperature of 370 °C, and a S-lens RF level of 30. Full MS settings were set to a resolution of 60,000, an AGC target of 1e6, a maximum injection time of 100 ms, and a scan range of 61 to 915 *m/z*. DDA settings were the following: resolution of 15,000, AGC target of 1e5, maximum injection time of 50 ms, isolation window of 1.0 m/z, stepped (N)CE of 20, 40, 60, minimum AGC target of 8e2, apex trigger of 1 to 4 s, charge exclusion ≥ 2, exclude isotopes on and dynamic exclusion of 7 s. During RP separation from 0 to 13 min, DDA was measured with top 5, and during HILIC separation from 13 to 25 min, DDA was measured with top 3. After injection of the extraction blank, an exclusion list using IE-Omics [32] was generated and used for standard and sample measurements. Furthermore, a sample pool was used for iterative injections with the generation of an iterative exclusion list using IE-Omics [32]. In each ionization mode, the sample pool was injected twice for the iterative workflow.

### Targeted data processing – Quantification of lactulose and mannitol

For quantification of lactulose and mannitol, the raw data was centroided and converted to.mzML files using MSConvert (version: 3.0.23051-d77d375), LC peak integration was done in Skyline (version: 23.1.0.268), and further data evaluation steps (QC inspection, blank subtraction, calculation of figures of merit, quantification of lactulose and mannitol using calibration curves, calculation of LMR, statistical analysis, and data visualization) were done in R studio (R version 4.3.2; R studio version 2024.04.2 + 764 “Chocolate Cosmos”). Details on software settings can be found in the Electronic Supplementary Material.

Based on figures of merit obtained during method validation (Fig. 2, Table S5), lactulose and mannitol were evaluated as M-H ions. Lactulose was measured in 1:10 diluted samples and mannitol in 1:100 diluted samples for the stand-alone HILIC method with Orbitrap ID-X detection. For the dual LC method with Orbitrap Q Exactive HF detection, both sugars were evaluated as M + Na adducts, and both sugars were measured in 1:50 diluted samples.

Quantification was done by normalizing peak areas of mannitol and lactulose to the peak area of the respective SIL internal standard. Using the area ratio, calibration curves over the whole calibration range were generated, and the working range (lower limit of quantification (LLOQ) and upper limit of quantification (ULOQ)) was automatically derived. Final quantification was done using sample-specific calibration curves using the 6 nearest calibration points compared to the sample.

For the assessment of intestinal permeability, the urinary excretion of lactulose and mannitol after 3 h of urine collection was calculated (Eq. 2: calculation of urinary sugar excretion) and the LMR, the ratio of lactulose and mannitol excretion (Eq. 3: lactulose:mannitol ratio (LMR)):

2 $$\%\;\mathrm{Sugar}\;\mathrm{excretion}=\frac{{\mathrm c}_\text{sugar}\text{ in urine }(\text{mg}/\text{mL})\ast{\mathrm V}_\text{urine}(\text{mL})}{{\mathrm m}_\text{sugar intake}(\text{mg})}\ast100$$

3 $$LMR=\frac{Lactulose\;recovery\;(\%)}{Mannitol\;recovery\;(\%)}$$

Statistical analysis to identify significant differences in the LMR between patients with major depressive disorder and the control group was performed using Welch’s two-sample *t*-test.

### Untargeted data processing for drug and drug metabolite monitoring

Untargeted data processing was done using Compound Discoverer 3.3 SP1 (Thermo Fisher Scientific). The workflow included two separate branches for the detection of compounds: (1) “Detect Compounds,” representing a typical metabolomics workflow, and (2) “Find Expected Compounds,” computing possible phase I and II biotransformation products for a given set of compounds (i.e., drugs listed in Table S7). The workflow tree is provided in the Electronic Supplementary Material (Figure S1). For further data evaluation steps within R studio, two excel files, one for each detection workflow, were exported from Compound Discoverer. Untargeted data was screened for drugs and their related metabolites. After manual filtering to confirm the identity of the drugs and their expected metabolites with identification confidence level 2 or 3 [33], the following data processing steps were undertaken: (1) the peak area was normalized to the creatinine concentration of each sample; (2) the normalized peak area for isomeric drug metabolites detected several times within one sample was summed up; (3) when a drug or metabolite was detected within both compound detection modes of Compound Discoverer, the mean value was calculated; and (4) finally for each drug and its related metabolite, the percentage of the total peak area (creatinine normalized) was calculated, which reflects the relative abundance of a drug and/or metabolite within a drug-metabolite set.

## Results and discussion

### Zwitterionic sulfobetaine stationary phase for separation of sugar isomers

**Fig. 1:**
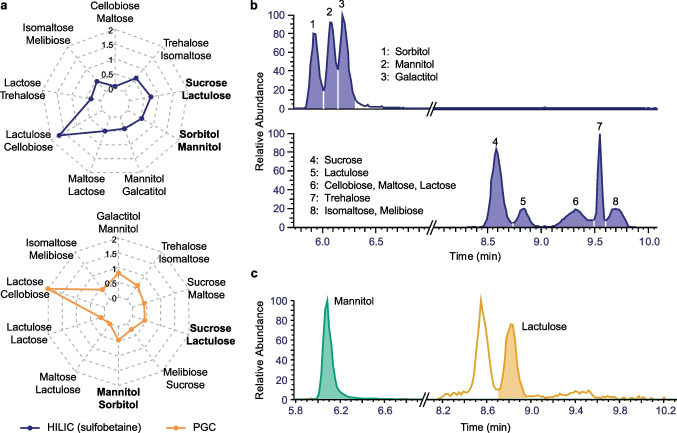
Separation of sugar isomers. **a** LC peak resolution of sugar alcohol and disaccharide isomers after the separation on a HILIC and PGC column. The resolution was calculated after the measurement of single standards using Eq. 1. **b** Chromatogram of sugar isomers within a multi-component standard using HILIC with zwitterionic sulfobetaine stationary phase. **c** Chromatogram of sugar isomers within a pooled urine sample after administration of lactulose and mannitol using HILIC with zwitterionic sulfobetaine stationary phase

The intestinal permeability test is an intervention-based assay, relying on the mass balance between the intake and excretion of the disaccharide lactulose and the sugar alcohol mannitol [22]. Thus, absolute quantification of the two sugar probes in urine is pursued, which requires the selective separation of their isomers. In fact, a survey of the urine metabolome database revealed the presence of several sugar alcohols and disaccharides in human urine [34], challenging the accurate quantification of lactulose and mannitol. Our method development focused on the sugar alcohol isomers mannitol, sorbitol, and galactitol, as well as the separation of the disaccharide isomers lactulose, sucrose, cellobiose, maltose, lactose, trehalose, isomaltose, and melibiose. For this panel of analytes, MS only strategies fail, as MS2 spectra are not selective. MS2 library spectra within the Massbank of North America (MoNA) measured with Orbitrap instruments do not show specific fragments for the two sugars of interest (Figure S2) [35]. Consequently, emphasis was placed on the separation of sugar isomers in the LC dimension. For the establishment of a suitable LC separation method, a sugar multi-component standard containing lactulose, mannitol, and their isomers, which are reported in the urine metabolome database [34], was prepared. Different stationary phases were tested including a HILIC column with a zwitterionic sulfobetaine phase, a HILIC column with an amide phase, a mixed mode column with RP and anion exchange capabilities, and a PGC column. The optimal separation conditions for each stationary phase (Table 1) were determined by testing a range of LC conditions and mobile phases. For the HILIC column with the amide stationary phase and the mixed mode column, no separation of sugar alcohol isomers was achieved. Hence, further method development was focused on the HILIC column with sulfobetaine stationary phase and the PGC column. The LC peak resolution between isomers on both columns is visualized in Fig. 1a (Table S2 and Table S3). Using these two columns, a similar separation of sugar alcohols was achieved. The three sugar alcohol isomers, which were analyzed in this study, were separated on both columns with a LC peak resolution between 0.4 and 0.8. For the disaccharide isomers, the separation on the sulfobetaine HILIC column was superior compared to the separation on the PGC column. Lactulose was separated from the two adjacent peaks representing sucrose and cellobiose with a resolution of 0.7 and 1.7, respectively. However, when using the PGC column, lactulose was not separated from maltose and lactose, and the separation of lactulose and sucrose was less effective with a resolution of 0.4 compared to the sulfobetaine HILIC column.

Overall, the HILIC method based on the zwitterionic sulfobetaine stationary phase provided the best performing separation when focusing on the separation of lactulose and mannitol from their respective isomers (Fig. 1b). Further validation steps focused on the separation of lactulose from sucrose as well as mannitol from sorbitol, as a preliminary screening of pooled patient urine samples narrowed down the panel of critical isomers (Fig. 1c). The accuracy of lactulose and mannitol quantification was assessed upon spiking the interfering isomers sucrose and sorbitol in concentrations covering three orders of magnitude (Figure S3). When lactulose and mannitol were present at concentrations > 1 µM, accurate quantification was achieved regardless of the concentration of isomeric sugars. The absence of baseline separation affected only the accuracy at sub-µM concentrations of lactulose and mannitol, which is within a concentration range that is lower than concentrations observed in the herein study (Table S4).

### Validation of dual LC–MS method

**Fig. 2:**
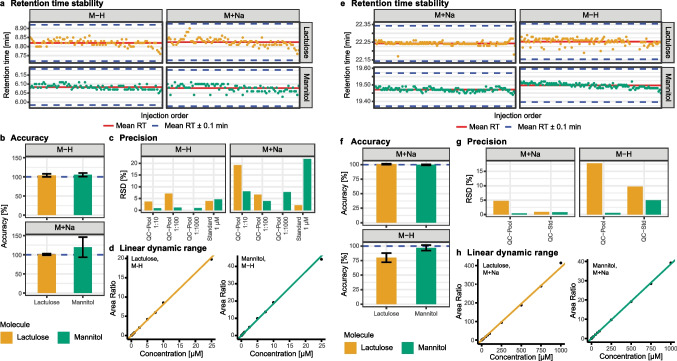
Retention time (RT) stability (**a**, **e**), accuracy (**b**, **f**), precision (**c**, **g**), and linear dynamic range (**d**, **h**) for quantification of lactulose and mannitol by LC–MS using HILIC separation with a sulfobetaine stationary phase. **a**–**d** Sugar quantification was done with stand-alone HILIC separation using MS detection by Orbitrap ID-X. **e**–**h** Sugar quantification was done within a dual LC setup combining RP and HILIC using MS detection by Orbitrap Q Exactive HF. For retention time stability assessment, the retention time was monitored either for 100 injections (**a**) or for 127 injections (**e**). The accuracy was calculated for repeated measurements (*N* = 9) of sugar standards at a target concentration of 1 µM (**b**) or 10 µM (**f**). The precision is expressed as the relative standard deviation (RSD) of repeated measurements (*N* = 9) of different QC samples (**c**, **g**). The linear dynamic range for both sugars was determined form calibration curves either in negative ionization mode as M-H ions (**d**) or in positive ionization mode as M + Na adducts (**h**)

The focus of this study was not only set on the targeted measurement and accurate quantification of mannitol and lactulose for intestinal permeability testing but also on the untargeted measurement of the same sample for drug monitoring. Therefore, the targeted and untargeted approach were merged in a dual LC–MS setup as previously shown [31]. To ensure the efficacy of the targeted HILIC method within the dual LC configuration, a comprehensive method validation was undertaken for both the stand-alone HILIC method and the dual LC method. The following setups were used: (1) HILIC separation with MS detection by Orbitrap ID-X and (2) dual LC setup combining RP and HILIC with MS detection by Orbitrap Q Exactive HF. For both methods the RT stability, accuracy, precision, and linear dynamic range were determined (Fig. 2, Table S5). The retention time for the sugars exhibited excellent stability in both analytical setups (RSD < 0.5%), thereby demonstrating the robustness of the LC separation. Both accuracy and precision depend on the ionization mode and the formed adduct. In the first setup (Orbitrap ID-X detection), superior performance was observed for both sugars in negative ionization mode (M-H ions), and in the second setup (dual LC with Orbitrap Q Exactive HF detection), superior performance was achieved in positive ionization mode (M + Na adducts). Finally, the linear dynamic range for setup 1 in negative ionization mode was three orders of magnitude for both sugars (LLOQ: 0.01 µM, ULOQ: 25 µM), and for setup 2 in positive ionization mode five orders of magnitude also for both sugars (LLOQ: 0.01 µM, ULOQ: 1000 µM). The expanded dynamic range with a higher ULOQ in setup 2 is because of the inclusion of more calibration points in the higher concentration range during validation experiments. These results confirm that both setups are fit for purpose for the quantification of lactulose and mannitol and thus suitable for intestinal permeability testing.

### Proof of principle: application of the dual LC–MS method

The power of integrating targeted assays into untargeted workflows especially in the clinical context was showcased in a proof of principle study on patients with major depressive disorder. This preliminary study analyzing five patient samples sets the frame for a future large-scale study on treatment-resistant depression. Increased intestinal permeability has been hypothesized to play a role in the development of depression [36–38]. Integrating the quantitative assay for intestinal permeability testing into a workflow of untargeted analysis offered to screen for drugs as confounding factors in clinical studies. Compliance to the therapeutic regime is of utmost importance in any clinical study [39]. This is even more important in the context of intestinal permeability testing as it is known that certain NSAIDs can affect test results [18, 19]. By integrating the targeted HILIC method for sugar probe quantification with an untargeted LC–MS method using RP separation in a dual LC setup, comprehensive data can be gathered on patients undergoing the test. The RP method enables untargeted metabolomics as well as the detection of exogenic compounds, such as drugs and their metabolites, following phase I and II biotransformation. If these substances undergo renal excretion, they can be detected in the urine samples collected during the intestinal permeability test, eliminating the need for additional sampling. The untargeted LC–MS approach allows for unbiased monitoring of patient drug compliance. The proof of principle study focused on patient samples rather than control samples, given the greater variety of potentially detectable drugs.

#### Targeted assay: quantification of lactulose and mannitol

**Fig. 3:**
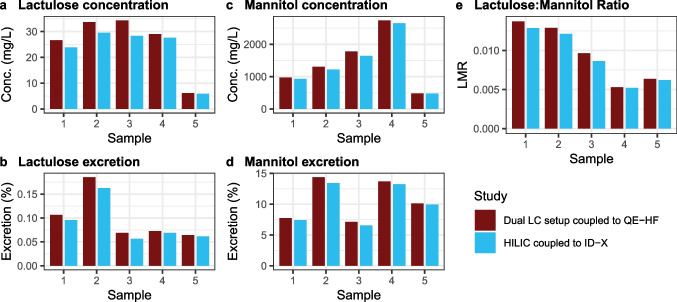
Quantification results for lactulose and mannitol following their oral administration for the assessment of intestinal permeability for five representative patient samples. Measurements were obtained using two independent methods: a dual LC setup coupled to an Orbitrap Q Exactive HF (red, setup 2) and stand-alone HILIC coupled to an ID-X (blue, setup 1). **a** and **c** show the measured concentration (mg/L) of lactulose and mannitol in urine, **b** and **d** display the excretion (%) of each sugar in urine, and **e** presents the lactulose:mannitol ratio (LMR)

Figure 3 (Table S6) compares absolute concentrations of lactulose and mannitol obtained in five patients by both LC setups. Here, we focus on concentration obtained with setup 2 (dual LC coupled to Orbitrap Q Exactive HF). The mean mannitol concentration normalized to creatinine within the study was 1463.0 ± 646.9 µmol/mmol creatinine. Considering that this concentration was around two orders of magnitude higher compared to reported sorbitol concentrations for adults in urine deposited in the Human Metabolome Database (HMDB) [40] (3.9 [1.9–5.1] µmol/mmol creatinine; 9.9 [2.5–18.7] µmol/mmol creatinine; 3.5 ± 2.24 µmol/mmol creatinine), these results confirm that the sorbitol interference for mannitol in this study would not introduce a trueness bias. The mean lactulose concentration normalized to creatinine within the study is 14.6 ± 9.2 µmol/mmol creatinine. When comparing these concentrations to the normal urinary sucrose levels reported in the HMDB for adults [40] (7.12 ± 3.23 µmol/mmol creatinine; 4.61 [0–9.21] µmol/mmol creatinine; 7.4 [1.4–19.5] µmol/mmol creatinine), given the on average higher mean concentration of lactulose and the accomplished peak resolution between sucrose and lactulose, a potential bias due to sucrose was excluded. For samples showing lactulose concentrations < 1 µM, manual peak inspection confirmed that the degree of peak overlap was minimal, ensuring that the quantification accuracy was still fit for purpose.

Based on the absolute quantification of lactulose and mannitol, the LMR, a measure of intestinal permeability [23], was assessed (Fig. 3, Table S6). The mean LMR was found to be 0.010 ± 0.004 (*N* = 5) for patients with major depressive disorder. This finding is consistent with the mean LMR of 0.009 ± 0.003 (*N* = 5) obtained for the same patient samples when measured independently with the stand-alone HILIC method (Fig. 3, Table S6). The mean LMR for a preliminary control group was found to be within the same range, at 0.011 ± 0.005 (*N* = 5) (Table S7). Thus, there was no significant difference between both groups (*p* = 0.6211).

#### Untargeted assay: drug monitoring

**Fig. 4:**
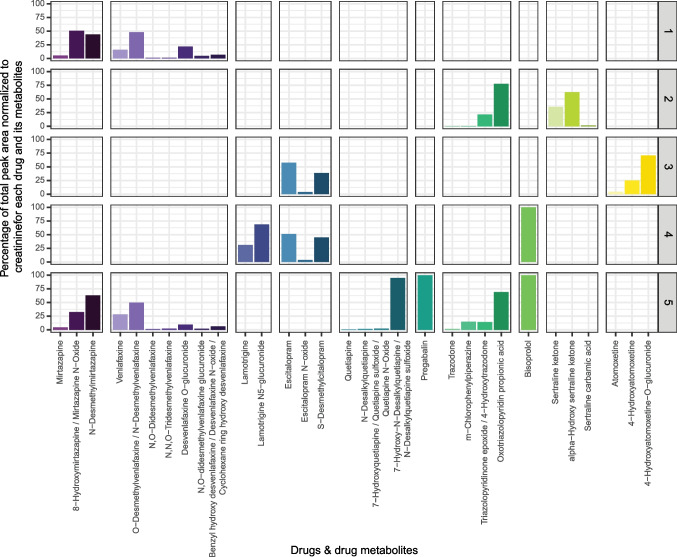
Detected drugs and their related metabolites by untargeted LC–MS/MS analysis. For each drug and its related metabolite, the percentage of the total peak area normalized to the creatinine concentration is shown. The percentages reflect the relative abundance of each drug and/or metabolite in relation to the total peak area (creatinine normalized) of a drug-metabolite-set. Each row represents a different patient sample

In the following, the utility of merging targeted with untargeted analysis in one workflow is addressed. Integrating the discovery approach, biologically interfering NSAIDs, that can increase intestinal permeability [18, 19], could be excluded. After suspect screening for NSAIDs (i.e., naproxen, indomethacin, meloxicam, and aspirin) based on their molecular formula and accurate mass, none of these could be detected on identification level 2 after matching against MS2 spectra of the mzCloud library in both patient and control samples. Furthermore, it was examined if patients did follow their medication regimen, which is documented in Table S8 together with drug metabolites reported in literature. Figure 4 shows the drugs and the metabolites for each patient, which were detected in positive ionization mode with an identification confidence level 2 or 3 [33] after suspect screening based on molecular formula and accurate mass as well as MS2 spectra matching against the mzCloud library. For the majority of the expected drugs, either the drug itself or drug metabolites were detected in the expected patient samples, confirming the patient compliance. However, for two drugs, levothyroxine and aripiprazole, neither the drug nor their metabolites were detected. The daily levothyroxine dosage for patient 4 was 0.1 mg, which is relatively low compared to other drugs administered in this study (Table S8). Thus, the absence of levothyroxine and its metabolites in the obtained results could be attributed to sensitivity limitations of the current LC–MS and data processing workflow. Patient 5 had a daily aripiprazole dosage of 10 mg, which, although higher than the levothyroxine dosage, remains on the lower end compared to other drug dosages within the study (Table S8). Additionally, since aripiprazole is primarily excreted through feces [41], its absence, along with that of its metabolites, could also be attributed to sensitivity limitations. To conclusively determine whether the method has sensitivity limitations for levothyroxine and aripiprazole or if the drugs were not detected due to non-adherence to the medication regimen, it would be necessary to measure standards for verification of detection limits.

For drugs and their metabolites, which are taken by multiple patients and for which more than one related compound can be detected (i.e., mirtazapine, venlafaxine, escitalopram, and trazodone), a comparison between the percentage of each drug and its metabolite based on their total peak area normalized to the creatinine concentration is possible (Fig. 4). Venlafaxine and escitalopram are metabolized similarly by the respective patients. For mirtazapine and trazodone, differences in the share of detected metabolites can be observed. However, for all patients who have been prescribed these drugs, the same metabolites, in addition to the drug itself, can be detected. These results could indicate differences in the biotransformation rate of these metabolites among different patients. To confirm such findings, the quantification of drugs and metabolites as well as a detailed pharmacokinetic analysis would be necessary.

### Intestinal permeability testing: harmonization issues

To establish the intestinal permeability test as a clinical diagnostic test, not only the integration of drug monitoring can improve its accuracy but in general harmonization of the testing procedure and its experimental design would be required. The comparison of urinary excretion (%) of lactulose and mannitol as well as the LMR between published studies is challenging because the intestinal permeability test using the two sugar probes is not standardized. Table 2 summarizes a selection of publications reporting the sugar excretion and/or LMR for healthy adult subjects. The analysis of the test design of the different studies indicates that both the amount of orally administered sugars and the time of urine collection differ. The amount of administered lactulose is between 5 and 10 g and mannitol between 0.5 and 10 g. The duration of urine collection varies between 2 and 8 h, with 5- and 6-h collection time being the most commonly used durations. In addition to administering lactulose and mannitol, a few studies also include sucralose that is used to investigate the large bowel permeability besides the small intestine permeability [42–44]. In the herein study, 10 g lactulose and 5 g mannitol were administered, and the urine collection time was 3 h. Despite differences in test design, a comparison is presented here to contextualize the obtained test results. A comparison to patients with major depressive disorder is not feasible, as no comparable study exists that has employed lactulose and mannitol for the assessment of intestinal permeability. The reported mannitol excretion, an indicator of the absorptive surface area [23], is 12–17% in most of the considered studies with two exceptions that report values of < 1% [42, 45]. In the five patients examined more closely in this study, the mannitol excretion ranges from 5 to 15% (Fig. 3d) and for controls from 7 to 11%. The lactulose excretion, an indicator of intestinal permeability [23], is reported with mean and median values ranging from 0.15 to 0.335%. The lactulose excretion measured in this study for patient samples ranges from 0.064 to 0.185% (Fig. 3b) and for controls from 0.066 to 0.147%. Mannitol and lactulose excretion are both lower for the herein examined patient and control samples compared to literature values for healthy adults, which may be attributable to the shorter urine collection time of 3 h, as compared to the 5–6 h used in most studies. Finally, the mean and median values of LMR, also an indicator of intestinal permeability [23], are reported between 0.012 and 0.0317, excluding the two studies with low mannitol excretion, which reported higher LMRs [42, 45]. A LMR of about 0.03 is often considered as cut-off value for an altered intestinal permeability [22, 45–48]. The LMRs of patients with major depressive disorder in this study (LMR: 0.005–0.014; *N* = 5) fall within the range of healthy adults according to this threshold. However, due to the lack of harmonization, interpretation of the data regarding intestinal permeability is inherently unreliable at *N* = 5. Consequently, all studies aiming to assess intestinal permeability must include a control group of healthy individuals. The LMRs for healthy controls in this study are between 0.007 and 0.019 (*N* = 5). Whether the quantitative LMR range is significantly different in patients compared to healthy controls can only be assessed by increasing the study size and stringent design of the healthy control cohort (e.g., matching age and sex).

**Table 2:** Literature values for lactulose and mannitol excretion (%) and LMR after intestinal permeability test within healthy adult subjects

Oral administration of sugars	Urine collection time	Mannitol % excretion*	Lactulose % excretion*	LMR*	Reference
5 g lactulose 2 g mannitol	5 h	12.300 (range 1.4800–43.7500	0.3550 (range 0.0204–1.8030)	0.0317 (range 0.0029–0.2510)	[22]
8 g lactulose 4 g mannitol	6–8 h (over-night collection)	Not reported	Not reported	0.030 (range 0.004–0.063)	[46]
10 g lactulose 0.5 g mannitol	6 h	15.9 ± 0.88	0.15 ± 0.01	Not reported	[49]
5 g lactulose 5 g mannitol	5 h	14.34 ± 0.33	0.3 ± 0.02	0.021 ± 0.001	[50]
7.5 g lactulose 2 g mannitol 2 g sucralose	5 h	0.53 ± 0.28	0.19 ± 0.22	0.27 ± 0.19	[42]
10 g lactulose 5 g mannitol 40 g sucrose	5 h	17.16 ± 1.57	0.21 ± 0.02	0.012 ± 0.001	[48]
5 g lactulose 2 g mannitol	2 h	0.085 (IQR 0.06–0.12)	0.26 (IQR 0.09–0.6)	0.062 (IQR 0.032–0.170)	[45]
10 g lactulose 10 g mannitol 10 g sucralose	5 h	Not reported	Not reported	0.023 (IQR 0.008–0.045)	[43]
5 g lactulose 2 g mannitol	5 h	Not reported	Not reported	0.027 (IQR 0.021–0.045)	[51]
5 g lactulose 10 g mannitol 40 g sucralose	6 h	Not reported	Not reported	0.013 (IQR 0.01–0.025)	[44]
5 g mannitol 10 g lactulose	5 h	12.71 ± 1.18	0.22 ± 0.02	Not reported	[47]

*Lactulose % excretion, mannitol % excretion, and lactulose:mannitol ratio (LMR) is either reported as mean ± SD or as median with range or interquartile range (IQR)

## Conclusion

In this study, a robust HILIC method utilizing a zwitterionic sulfobetaine stationary phase was developed and validated for the separation and quantification of lactulose and mannitol. The method development, investigating different stationary phases, focused on the separation of these sugars from their respective isomers. The HILIC method was integrated with a RP method in a dual LC–MS workflow that combines targeted sugar quantification with untargeted analysis of exogenic substances (i.e., drugs and their metabolites). This workflow was applied to urine samples from patients with major depressive disorder undergoing intestinal permeability testing, confirming the method’s suitability for assessing intestinal permeability using lactulose and mannitol as probe sugars. The mean LMR was in the same range for depressed individuals as for healthy individuals. Additionally, the integration of the untargeted analysis provided valuable insights into patient drug compliance, demonstrating the method’s versatility in clinical research studies. By integrating the untargeted analysis based on a metabolomics workflow, the whole workflow can be expanded to a holistic untargeted metabolomics analysis giving valuable insights into patients’ disease status within clinical studies. This study offers a comprehensive analytical workflow for both targeted and untargeted analysis of patient urine samples in the context of intestinal permeability testing. Differences between target groups or intra-individual changes under different treatment strategies will be investigated in larger sample sizes.

## Supplementary Information

Below is the link to the electronic supplementary material.

Supplementary file1 (PDF 489 KB)
