# Supplementary material for: Integrating the lactulose-mannitol test for intestinal permeability with untargeted metabolomics for drug monitoring through dual liquid chromatography-mass spectrometry
Source: Anal Bioanal Chem. 2025 Feb 27;417(13):2767–81. doi: 10.1007/s00216-025-05790-7 (PMC12053204; doi:10.1007/s00216-025-05790-7)
Supplement: Supplementary file 1 — Supplementary file1 (PDF 489 KB) [file 216_2025_5790_MOESM1_ESM.pdf]

Supporting Information

Integrating the lactulose-mannitol test for intestinal permeability with untargeted metabolomics for drug monitoring through dual liquid chromatography-mass spectrometry

Felina Hildebrand[1,2], Cemre Cukaci[3,4], Harald Schoeny[1], Christoph Baumgartinger[1], Bruno Stelzer[1], Matteo Spedicato[5], Tobias Frey[6], Martina Catani[5], Klaus Schmetterer[6], Richard Frey[3,4], Gunda Koellensperger[1,7]*

[1] Department of Analytical Chemistry, University of Vienna, Waehringer Str. 38, 1090 Vienna, Austria

[2] Vienna Doctoral School in Chemistry (DoSChem), University of Vienna, Waehringer Str. 42, 1090 Vienna, Austria

[3] Division of General Psychiatry, Department of Psychiatry and Psychotherapy, Medizinische Universität Wien, Wien, Österreich / Medical University of Vienna, Vienna, Austria

[4] Comprehensive Center for Clinical Neurosciences and Mental Health, Medizinische Universität Wien, Wien, Österreich / Medical University of Vienna, Vienna, Austria

[5] Department of Chemical, Pharmaceutical and Agricultural Sciences – DOCPAS, University of Ferrara, Via L.Borsari 46, 44121 Ferrara (ITALY)

[6] Department of Laboratory Medicine, Medical University of Vienna, Waehringer Guertel 18-20, 1090 Vienna, Austria

[7] Vienna Metabolomics Center (VIME), University of Vienna, Althanstr. 14, 1090 Vienna, Austria

* Corresponding author: Gunda Koellensperger (gunda.koellensperger@univie.ac.at)

Table of Contents

Supporting methods 2

Solvents and standards 2

Assessment of isomeric interferences 2

Sample preparation 3

Targeted data evaluation 4

Supporting figures 4

Supporting tables 6

References 9

Supporting methods

Solvents and standards

All solvents (water, acetonitrile, and methanol) were LC-MS grade and ordered at Honeywell (Vienna, Austria). Formic acid was purchased from VWR International (Vienna, Austria), ammonium hydroxide solution (25-27%) from Honeywell (Vienna, Austria) and ammonium bicarbonate from Merck (Vienna, Austria.)

Sugar standards (D-Mannitol, Lactulose, D-Mannitol-^{13}C6, Lactulose-^{13}C12, Galactitol, D-Sorbitol, D-(+)-Melibiose, D-(+)-Cellobiose, D-(+)-Maltose, α-Lactose, Sucrose, Isomaltose, and Trehalose) were ordered at Sigma Aldrich or Merck (Vienna, Austria).

Metabolite standards were obtained from Sigma Aldrich (Vienna, Austria) and Carbosynth (Berkshire, UK). Each compound was weighed, dissolved, and then combined to create an equimolar mixture of 147 metabolite standards. The solvent was subsequently removed by vacuum centrifugation, and the dried standards were stored in HPLC vials at -80 °C. For untargeted measurements, this standard mix was included and measured at a concentration of 1 µM.

Assessment of isomeric interferences

To assess the isomeric interferences of other sugars mixes with sugars at different concentrations were quantified via a calibration curve with 13 calibration points (0.001 µM, 0.01 µM, 0.05 µM, 0.1 µM, 0.25 µM, 0.5 µM, 0.75 µM, 1 µM, 2.5 µM, 5 µM, 7.5 µM, 10 µM, and 25 µM) and ISTD concentrations of 1 µM for lactulose-^{13}C12 and 0.5 µM for D-mannitol-^{13}C6 prepared in 80:20 ACN/water (v/v).

The in the main manuscript describe HILIC separation based on a sulfobetaine stationary phase was utilized as a stand-alone separation with a Q Exactive HF™ quadrupole-Orbitrap mass spectrometer (Thermo Fisher Scientific) as detector. The injection volume was 5 µL. The autosampler and column temperature were constantly maintained at 6 °C and 40 °C, respectively. Ionization after LC separation was achieved using heated electrospray ionization (HESI) with the following parameters: spray voltage of 3.5 kV (+) and 2.8 kV (-), capillary temperature of 280 °C, sheath gas flow rate of 40, aux gas flow rate of 3, sweep gas flow rate of 0, heater temperature of 320 °C and a S-lens RF level of 50. Data acquisition was done with polarity switching in MS1 mode with a resolution of 60000, an AGC target of 1e6, a maximum injection time of 100 ms, and a scan range of 60 to 900 *m/z* in positive mode and 63 to 945 *m/z* in negative mode.

Quantification was done based on M-H ions after peak integration in Skyline.

Sample preparation

For extraction in microcentrifugation, 200 µL urine (or water as extraction blank) was mixed with 10 µL of internal standard (ISTD) solution (0.2 mM lactulose-^{13}C12 and 10 mM D-mannitol-^{13}C6) and 840 µL MeOH. After incubation for 1.5 h at -20 °C samples were centrifuged for 15 min at 14000 g and 4 °C, the supernatant was transferred into a new microcentrifuge tube and dried. For measurement the samples were reconstituted in 200 µL water, ultra-sonicated for 1 min, and filtered (Claristep Syringeless Filters, 0.2 µm, Sartorius). 20 µL of each extract was taken to create a quality control (QC) pool. Finally, samples and the QC pool were diluted 1:10, 1:100, and 1:1000 all in 80:20 ACN/water (v/v) and measured by HILIC coupled to Orbitrap ID-X.

For the down-scaled extraction in a 96-well, 40 µL of urine (or water as extraction blank) was mixed with 210 µL of the extraction solvent (200 µL MeOH and 10 µL ISTD mixture containing 5 mM D-mannitol-^{13}C6 and 0.5 mM lactulose-^{13}C12). After shaking for 3 min at 500 rpm and 4 °C extracts were incubated for 1.5 h at -20 °C. Afterward the samples were centrifuged for 15 min at 4500 rpm and 4 °C and the whole supernatant was transferred to a new 96-well plate. Finally, the samples were evaporated to dryness and stored at -20 °C until measurement. For measurement samples were dissolved in 40 µL water, ultra-sonicated for 3 min, and centrifuged for 15 min at 4500 rpm and 4 °C. For measurement samples were diluted 1:50 in 80:20 ACN/water (v/v) and injected on the HILIC column within a dual LC setup coupled to Orbitrap Q Exactive HF.

For untargeted measurements, the down-scaled extraction procedure was followed without spiking of stable isotope labeled internal standards. However, instead of internal standards water was added to the extraction solvent. Extracts for untargeted measurement were 1:10 diluted in water and injected on the RP column within a dual LC setup coupled to Orbitrap Q Exactive HF.

For measurements on Orbitrap ID-X, a calibration curve with 13 calibration points (0.001 µM, 0.01 µM, 0.05 µM, 0.1 µM, 0.25 µM, 0.5 µM, 0.75 µM, 1 µM, 2.5 µM, 5 µM, 7.5 µM, 10 µM, and 25 µM) and a ISTD concentration of 1 µM for lactulose-^{13}C12 and 0.5 µM for D-mannitol-^{13}C6 was prepared in 80:20 ACN/water (v/v).

For measurement on Orbitrap Q Exactive HF, a calibration curve dissolved in 80% ACN with 18 calibration points at equimolar concentrations of D-mannitol and lactulose and ISTD concentration of 25 µM for D-mannitol-^{13}C6 and 2.5 µM for lactulose-^{13}C12 was prepared. The

following standard concentrations were prepared: 0.01 µM, 0.05 µM, 0.1 µM, 0.5 µM, 0.75 µM, 1 µM, 2.5 µM, 5 µM, 7.5 µM, 10 µM, 25 µM, 50 µM, 75 µM, 100 µM, 250 µM, 500 µM, 750 µM, and 1000 µM.

Targeted data evaluation

For data measured on Orbitrap ID-X, MSConvert (Version: 3.0.23051-d77d375) was used with peak picking set to vendor msLevel=1-1 and for data measured in the dual LC setup, MSConvert (Version: 3.0.23051-d77d375) settings were the following: peak picking was set to vendor msLevel=1-2 and scan Time was set to [780,1500]. Skyline (Version: 23.1.0.268) was used with the following MS1 filtering settings: Isotope peaks included set to Count, Precursor mass analyzer set to Centroided, Peaks is 1 and Mass Accuracy 5 ppm.

Supporting figures

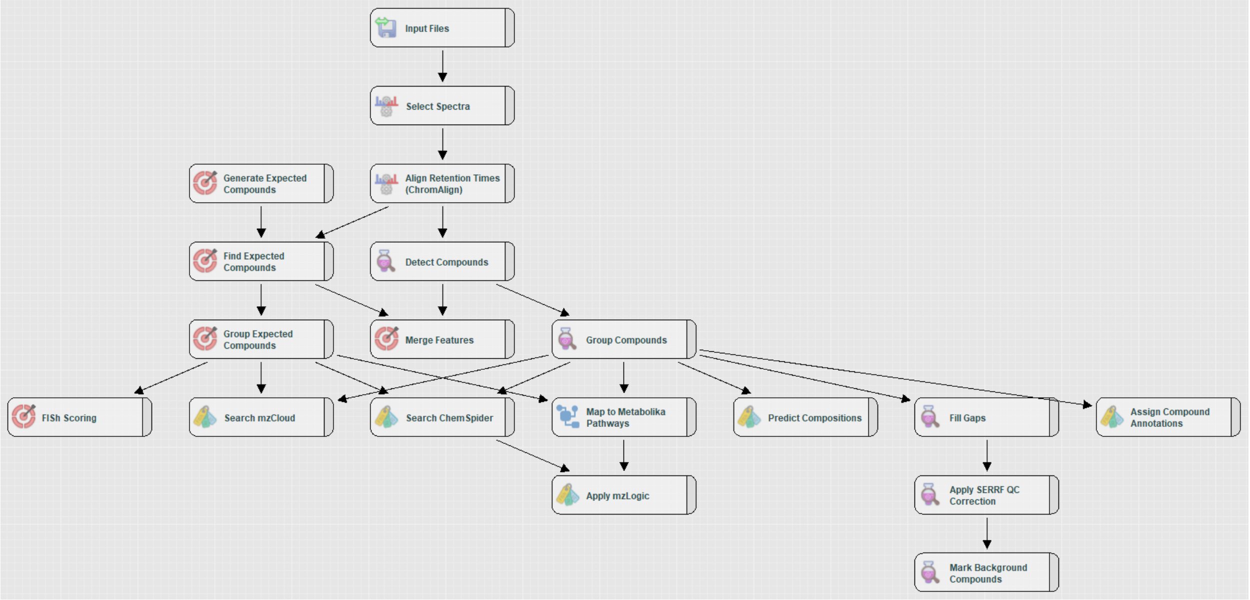

Figure S1: Workflow tree for untargeted metabolomics data processing together with biotransformation workflow for drugs (expected compounds) in Compound Discoverer.

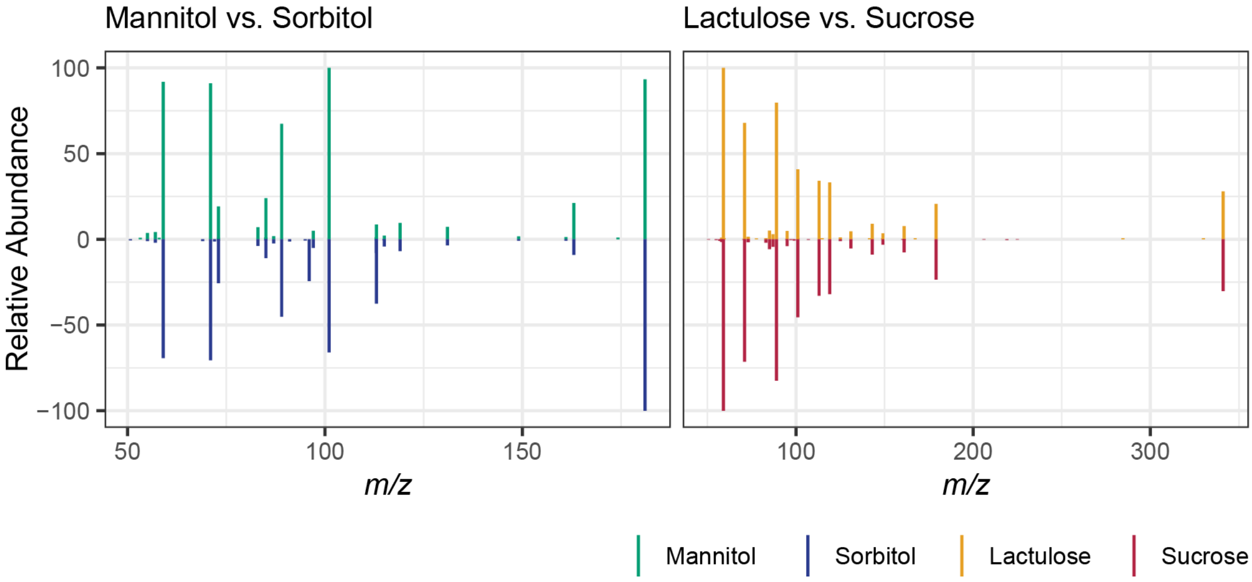

Figure S2: MS2 library spectra taken from Massbank of North America (MoNA) [1] for mannitol, sorbitol, lactulose, and sucrose. Spectra of isomers are shown as mirror spectra.

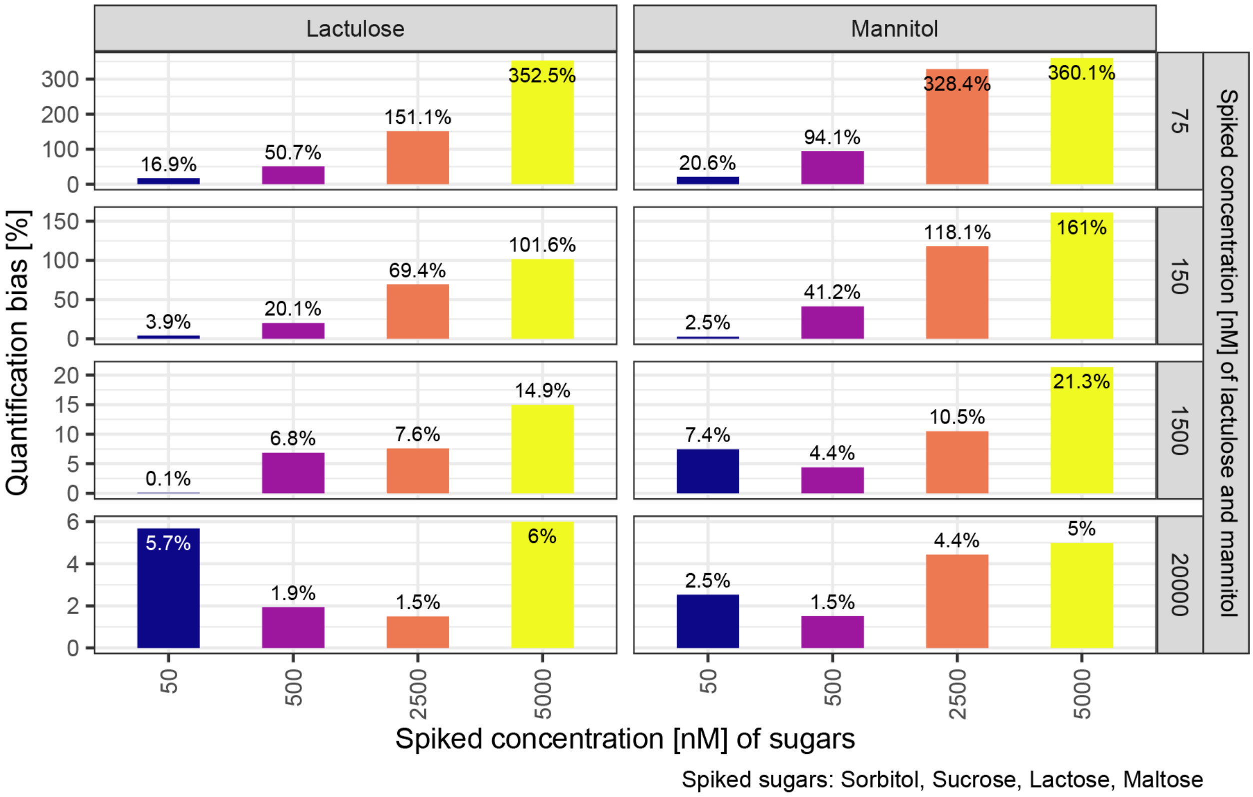

Figure S3: Quantification bias [%] for lactulose and mannitol at different concentrations (75 nM, 150 nM, 1.5 µM, and 20 µM) when spiking with isomeric compounds (sorbitol, sucrose, lactose, and maltose) at different concentrations (50 nM, 500 nM, 2.5 µM, 5 µM).

Supporting tables

Table S1: Metadata (sex and age) for analyzed patient with major depressive disorder and control samples

Sample number	Patient/ Control	Sex	Age
1	Patient	Male	37
2	Patient	Male	23
3	Patient	Female	18
4	Patient	Female	65
5	Patient	Male	44
6	Control	Male	31
7	Control	Male	25
8	Control	Female	22
9	Control	Female	61
10	Control	Male	52

Table S2: LC peak resolution for HILIC (sulfobetaine). LC peak detection was done manually using FreeStyle after measuring single standards (c = 5 µM) using Q Exactive HF in negative ionization mode.

Molecule class	Molecule	RT (min)	Baseline width (min)	Peak pair	Resolution
Sugar alcohol	Sorbitol	5.75	0.26	Sorbitol - Mannitol	0.5
Sugar alcohol	Mannitol	5.91	0.31	Mannitol - Galcatitol	0.4
Sugar alcohol	Galactitol	6.03	0.26		
Disaccharide	Sucrose	8.26	0.35	Sucrose - Lactulose	0.7
Disaccharide	Lactulose	8.52	0.35	Lactulose - Cellobiose	1.7
Disaccharide	Cellobiose	9.16	0.40	Cellobiose - Maltose	0.1
Disaccharide	Maltose	9.18	0.44	Maltose - Lactose	0.5
Disaccharide	Lactose	9.36	0.26	Lactose - Trehalose	0.3
Disaccharide	Trehalose	9.43	0.16	Trehalose - Isomaltose	0.6
Disaccharide	Isomaltose	9.55	0.22	Isomaltose - Melibiose	0.5
Disaccharide	Melibiose	9.65	0.23		

Table S3: LC peak resolution for PGC. LC peak detection was done manually using FreeStyle after measuring single standards (c = 5 µM) using Q Exactive HF in negative ionization mode.

Molecule class	Molecule	RT (min)	Baseline width (min)	Peak pair	Resolution
Sugar alcohol	Galactitol	1.17	0.12	Galactitol - Mannitol	0.8
Sugar alcohol	Mannitol	1.27	0.13	Mannitol -Sorbitol	0.5
Sugar alcohol	Sorbitol	1.34	0.15		
Disaccharide	Trehalose	4.79	0.08	Trehalose - Isomaltose	0.6
Disaccharide	Isomaltose	4.85	0.12	Isomaltose - Melibiose	0.4
Disaccharide	Melibiose	4.91	0.17	Melibiose - Sucrose	0.2
Disaccharide	Sucrose	4.94	0.08	Sucrose - Maltose	0.4
Disaccharide	Maltose	4.98	0.09	Maltose - Lactulose	0.0
Disaccharide	Lactulose	4.98	0.08	Lactulose - Lactose	0.1
Disaccharide	Lactose	5.00	0.15	Lactose - Cellobiose	2.0
Disaccharide	Cellobiose	5.24	0.10		

Table S4: Lactulose and mannitol concentrations (µM) measured in diluted urine extracts of patients by two different LC-MS setups. For setup 1 lactulose was measured in a 1:10 dilution and mannitol in a 1:100 dilution. For setup 2 both sugars were measured in a 1:50 dilution.

Analytical setup	Sample	Measured lactulose concentration in diluted extract (µM)	Measured mannitol concentration in diluted extract (µM)
Setup 1: HILIC separation with MS detection by Orbitrap ID-X	1	6.98	5.10
	2	8.64	6.70
	3	8.27	8.99
	4	8.06	14.54
	5	1.71	2.60
Setup 2: Dual LC with MS detection by Orbitrap Q Exactive HF	1	1.55	106.44
	2	1.97	143.35
	3	2.01	195.18
	4	1.70	300.58
	5	0.36	52.91

Table S5: HILIC method validation (RT stability, accuracy, precision, linear dynamic range) for the quantification of lactulose and mannitol using two different analytical setups.

Analytical setup	Molecule	Adduct	Retention time stability	Accuracy	Precision	Linear dynamic range
Setup 1: HILIC separation with MS detection by Orbitrap ID-X	Lactulose	M+Na	0.24%	100.7% ± 2.2%	Standard (1 µM): 2.2%<br>QC-Pool (1:10): 19.3%<br>QC-Pool (1:100): 6.7%	LLOQ: 0.01 µM<br>ULOQ: 25µM
		M-H	0.20%	103.9% ± 4.1%	Standard (1 µM): 3.9%<br>QC-Pool (1:10): 3.7%<br>QC-Pool (1:100): 7.1%	LLOQ: 0.01 µM<br>ULOQ: 25µM
	Mannitol	M+Na	0.31%	119.6% ± 26.1%	Standard (1 µM): 21.9%<br>QC-Pool (1:10): 8.1%<br>QC-Pool (1:100): 4.0%<br>QC-Pool (1:1000): 7.8%	LLOQ: 0.05 µM<br>ULOQ: 25µM
		M-H	0.22%	105.3% ± 4.9%	Standard (1 µM): 4.7%<br>QC-Pool (1:10): 0.9%<br>QC-Pool (1:100): 1.2%<br>QC-Pool (1:1000): 1.0%	LLOQ: 0.01 µM<br>ULOQ: 25µM
Setup 2: Dual LC with MS detection by Orbitrap Q Exactive HF	Lactulose	M+Na	0.05%	100.9% ± 0.9%	Standard (10 µM): 0.9%<br>QC-Pool (1:50): 4.7%	LLOQ: 0.01 µM<br>ULOQ: 1000 µM
		M-H	0.08%	80.0% ± 7.8%	Standard (10 µM): 9.7%<br>QC-Pool (1:50): 17.8%	LLOQ: 0.5 µM<br>ULOQ: 1000 µM
	Mannitol	M+Na	0.06%	99.8% ± 0.8%	Standard (10 µM): 0.8%<br>QC-Pool (1:50): 0.4%	LLOQ: 0.01 µM<br>ULOQ: 1000 µM
		M-H	0.07%	96.9% ± 4.8%	Standard (10 µM): 4.9%<br>QC-Pool (1:50): 0.6%	LLOQ: 0.01 µM<br>ULOQ: 1000 µM

Table S6: Results of intestinal permeability test for patients with major depressive disorder determined by two different LC-MS setups.

Analytical setup	Molecule	Concentration (mg/L)	Urinary excretion	Lactulose:mannitol ratio (LMR)
**Setup 1: HILIC separation with MS detection by Orbitrap ID-X**	Lactulose	23.0 ± 9.8	0.089% ± 0.044%	0.009 ± 0.003
	Mannitol	1381.9 ± 825.8	10.1% ± 3.2%	
**Setup 2: Dual LC with MS detection by Orbitrap Q Exactive HF**	Lactulose	25.9 ± 11.5	0.099% ± 0.051%	0.010 ± 0.004
	Mannitol	1454.6 ± 859.5	10.6% ± 3.3%	

Table S7: Results of intestinal permeability test for control samples measured by dual LC with MS detection by Orbitrap Q Exactive HF

Molecule	Urinary excretion	Lactulose:mannitol ratio (LMR)
Lactulose	0.095% ± 0.034%	0.011 ± 0.005
Mannitol	9.0% ± 1.2%	

Table S8: Drugs, which are part of the medication regimen of selected patients, and the in literature reported metabolites. [2, 3]

Drug	Drug metabolites	Sample number (daily dosage of drug)
Mirtazapine (C17 H19 N3)	8-Hydroxymirtazapine (C17 H19 N3 O),<br>Mirtazapine N-Oxide (C17 H19 N3 O),<br>N-Desmethylmirtazapine (C16 H17 N3)	1 (90 mg)<br>5 (90 mg)
Venlafaxine (C17 H27 N O2)	Desvenlafaxine / O-Desmethylvenlafaxine (C16 H25 N O2),<br>N-Desmethylvenlafaxine (C16 H25 N O2),<br>N,O-Didesmethylvenlafaxine (C15 H23 N O2),<br>N,N,O-Tridesmethylvenlafaxine (C14 H21 N O2),<br>Desvenlafaxine O-glucuronide (C22 H33 N O8),<br>N,O-didesmethylvenlafaxine glucuronide (C21 H31 N O8),<br>Benzyl hydroxy desvenlafaxine (C16 H25 N O3),<br>Cyclohexane ring hydroxy desvenlafaxine (C16 H25 N O3),<br>Desvenlafaxine N-oxide (C16 H25 N O3)	1 (375 mg)<br>5 (450 mg)
Lamotrigine (C9 H7 Cl2 N5)	Lamotrigine N2-glucuronide (C15 H16 Cl2 N5 O6),<br>Lamotrigine N5-glucuronide (C15 H15 Cl2 N5 O6),<br>N2-Methyl lamotrigine (C10 H10 Cl2 N5)	4 (150 mg)
Escitalopram (C20 H21 F N2 O)	S-Desmethylcitalopram (C19 H19 F N2 O),<br>Escitalopram propionaldehyde (C18 H14 F N O2),<br>Escitalopram propionic acid (C18 H13 F N O3),<br>S-Didesmethylcitalopram (C18 H17 F N2 O),<br>Escitalopram N-oxide (C20 H21 F N2 O2)	4 (20 mg)<br>3 (10 mg)
Levothyroxine (C15 H11 I4 N O4)	Liothyronine (C15 H12 I3 N O4),<br>Reverse triiodothyronine (C15 H12 I3 N O4),<br>Diiodothyronine (C15 H13 I2 N O4),<br>Monoiodothyronine (C15 H14 I N O4),<br>Levothyroxine Acyl Glucuronide (C21 H19 I4 N O10),<br>Triiodothyronine sulfate (C15 H12 I3 N O7 S)	4 (0.1 mg)
Quetiapine (C21 H25 N3 O2 S)	N-Desalkylquetiapine (C17 H17 N3 S),<br>7-Hydroxyquetiapine (C21 H25 N3 O3 S),<br>Quetiapine sulfoxide (C21 H25 N3 O3 S),	5 (150 mg)

	Quetiapine N-Oxide (C21 H25 N3 O3 S),<br>O-Desalkylquetiapine (C19 H21 N3 O S),<br>7-Hydroxy-N-Desalkylquetiapine (C17 H17 N3 O S),<br>N-Desalkylquetiapine sulfoxide (C17 H17 N3 O S)	
Pregabalin (C8 H17 N O2)	N-Methylpregabalin (C9 H19 N O2)	5 (1200 mg)
Trazodone (C19 H22 Cl N5 O)	m-Chlorophenylpiperazine (C10 H13 Cl N2),<br>Triazolopyridinone dihydrodiol (C19 H24 O3 N5 Cl),<br>Triazolopyridinone epoxide (C19 H22 Cl N5 O2),<br>4-Hydroxytrazodone (C19 H22 Cl N5 O2),<br>Oxotriazolopyridin propionic acid (C9 H9 N3 O3)	5 (150 mg)<br>2 (100 mg)
Aripiprazole (C23 H27 Cl2 N3 O2)	Dehydroaripiprazole (C23 H25 Cl2 N3 O2),<br>Dehydroaripiprazole epoxide (C23 H23 Cl2 N3 O3),<br>Dehydro-aripiprazole unnamed glutathione conjugate 1 (C33 H40 Cl2 N6 O8 S),<br>Dehydro-aripiprazole unnamed glutathione conjugate 2 (C33 H40 Cl2 N6 O9 S),<br>4-Hydroxyaripiprazole (C23 H27 Cl2 N3 O3),<br>Aripiprazole unnamed metabolite 1 (C23 H26 Cl2 N3 O3),<br>Aripiprazole unnamed metabolite 2 (C33 H42 Cl2 N6 O9 S),<br>Aripiprazole unnamed metabolite 3 (C6 H2 Cl2 O2),<br>Aripiprazole unnamed metabolite 4 (C16 H17 Cl2 N3 O8 S),<br>2,3-Dichlorophenylpiperazine (C10 H12 Cl2 N2),<br>4-[(2-oxo-3,4-dihydro-1H-quinolin-7-yl)oxy]butanal (C13 H15 N O3),<br>Aripiprazole unnamed metabolite 5 (C23 H26 Cl2 N3 O2),<br>Aripiprazole unnamed metabolite 6 (C23 H26 Cl2 N3 O2),<br>Aripiprazole unnamed metabolite 7 (C23 H27 Cl2 N3 O3),<br>Aripiprazole unnamed metabolite 8 (C23 H27 Cl2 N3 O3),<br>Aripiprazole unnamed metabolite 9 (C33 H44 Cl2 N6 O9 S)	5 (10 mg)
Bisoprolol (C18 H31 N O4)		4 (2.5 mg)<br>5 (2.5 mg)
Sertraline (C17 H17 Cl2 N)	Norsertraline / Desmethylsertraline (C16 H15 Cl2 N),<br>Sertraline ketone (C16 H12 Cl2 O),<br>alpha-Hydroxy sertraline ketone (C16 H12 Cl2 O2),<br>alpha-Hydroxy sertraline ketone glucuronide (C22 H20 Cl2 O8),<br>Sertraline carbamic acid (C17 H15 Cl2 N O2),<br>Sertraline carbamoyl-O-glucuronide (C24 H25 Cl2 N O8)	2 (100 mg)
Atomoxetine (C17 H21 N O)	4-Hydroxyatomoxetine (C17 H21 N O2),<br>4-Hydroxyatomoxetine-O-glucuronide (C23 H29 N O8),<br>N-Desmethylatomoxetine (C16 H19 N O),<br>N-Desmethyl-4-hydroxyatomoxetine (C16 H19 N O2)	3 (25 mg)

References

1. MassBank of North America. https://mona.fiehnlab.ucdavis.edu/. Accessed 21 Mar 2022

2. Knox C, Wilson M, Klinger CM, Franklin M, Oler E, Wilson A, Pon A, Cox J, Chin NE (Lucy), Strawbridge SA, Garcia-Patino M, Kruger R, Sivakumaran A, Sanford S, Doshi R, Khetarpal N, Fatokun O, Doucet D, Zubkowski A, Rayat DY, Jackson H, Harford K, Anjum A, Zakir M, Wang F, Tian S, Lee B, Liigand J, Peters H, Wang RQ (Rachel), Nguyen T, So D, Sharp M, da Silva R, Gabriel C, Scantlebury J, Jasinski M, Ackerman D, Jewison T, Sajed T, Gautam V, Wishart DS (2024) DrugBank 6.0: the DrugBank

Knowledgebase for 2024. Nucleic Acids Research 52:D1265–D1275. https://doi.org/10.1093/nar/gkad976

3. Kim S, Chen J, Cheng T, Gindulyte A, He J, He S, Li Q, Shoemaker BA, Thiessen PA, Yu B, Zaslavsky L, Zhang J, Bolton EE (2023) PubChem 2023 update. Nucleic Acids Research 51:D1373–D1380. https://doi.org/10.1093/nar/gkac956
